# Identification of FGF21‐inducing rare sugars that reduces sugar appetite in male BL/6 mice

**DOI:** 10.14814/phy2.70618

**Published:** 2025-10-15

**Authors:** Oulan G. H. N. B. Efendi, Sho Matsui, Satoshi Tsuzuki, Tsutomu Sasaki

**Affiliations:** ^1^ Laboratory of Nutrition Chemistry, Division of Food Science and Biotechnology, Graduate School of Agriculture Kyoto University Kyoto Japan

**Keywords:** fibroblast growth factor 21, oxytocin neurons, rare sugars, sugar appetite

## Abstract

Dietary sugars induce the secretion of hepatic fibroblast growth factor 21 (FGF21) and subsequently activate FGF21‐sensing oxytocin neurons to suppress sugar intake. We aimed to identify FGF21‐inducing rare sugars as substitutes for obesogenic dietary sugars and to test their ability to suppress sugar appetite in BL/6 mice. We have identified D‐allulose, D‐tagatose, and D‐sorbitol as potent FGF21‐inducers in mouse primary hepatocytes. All three compounds were confirmed to induce FGF21 secretion and subsequently activate oxytocin neurons in mice. FGF21‐inducing rare sugars administered by gastric gavage to mice reduced sugar intake, and mixing these sugars with sucrose solution significantly reduced their intake and preference in mice. The long‐term lick analyses showed that an FGF21‐inducing sugar made the solution palatable, but reduced the appetite for the sugar solution and prolonged the ingestion interval in mice. Therefore, using these sugars as substitutes for obesogenic dietary sugars may help to curve sugar appetite and reduce sugar intake.

## Graphical abstract

Dietary sugars induced FGF21 secretion from the liver and activate hypothalamic oxytocin neurons to reduce furter sugar intake. Replacing obesogenic dietary sugars with FGF21‐inducing rare sugars can stimulate the same system and reduce sugar appetite.

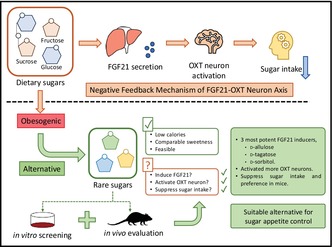

## 1 INTRODUCTION

Overeating due to an increased appetite is a major cause of obesity. While predisposing genetic variations may contribute to overeating, sugar consumption increases hunger sensations (Davis et al., 2011; Razzoli et al., 2017). A heightened sweet preference is reported to increase hypothalamic activation, which potentially disrupts appetite regulation and increases the risk of weight gain (Penaforte et al., 2013). Dietary restriction is an option to manage overeating; however, adherence is highly influenced by multiple factors (Hall et al., 2015; Liu et al., 2022; Reents & Pedersen, 2021). Understanding the mechanisms that reduce sugar intake is important for tackling overeating and obesity.

Fibroblast growth factor 21 (FGF21) is one of the negative feedback endocrine signals that regulate sugar intake (von Holstein‐Rathlou & Gillum, 2019). It is a hepatokine that regulates body homeostasis in response to different macronutrient balances and fasting (Hill et al., 2020; Liang et al., 2014). FGF21 activates neuronal subpopulations that express FGFR1/β‐Klotho heterodimer receptor complex, which are located in various brain regions (Lan et al., 2017). Oxytocin neurons of the paraventricular nucleus of the hypothalamus (PVH^Oxt^) have been identified as a target of FGF21 for sucrose preference suppression, forming a negative feedback mechanism in the liver‐brain axis (Matsui et al., 2018). Because sucrose as dietary sugar is obesogenic, a low‐calorie natural sweetener that potentially activates this axis could serve as an alternative for sugar appetite suppression.

Rare sugars, a group of monosaccharides and their derivatives with low abundance in nature, are poorly metabolized in the body and have negligible amounts of calories but comparable sweetness to dietary sugars (Ahmed et al., 2021). Although mechanistically elusive, adding rare sugars to the diet has been reported to reduce food intake in both rodents and humans (Hayashi et al., 2014; Nagata et al., 2017). We have hypothesized that this reduction in food intake may be mediated through the activation of FGF21 the PVH^Oxt^. In this study, we aimed to identify FGF21‐inducers among rare sugars and evaluate their efficacy in controlling sugar appetite in male mice.

## 2 MATERIALS AND METHODS

### 2.1 Animals

Male wild‐type C57BL/6 mice (CLEA Japan, Tokyo, Japan) weighing between 21 and 23 g were used for investigations in this study. Mice were individually kept in sterilized cages under a 12‐h light–dark cycle with controlled temperature and humidity. Normal chow (NC) diet (Oriental Yeast Co., Ltd., Tokyo, Japan) and water were provided ad libitum until use. The nutritional composition of the NC diet is summarized in Table S1. All of the experiments were conducted in accordance with the Regulations of Animal Experimentation at Kyoto University (approval number: 31‐75 and R2‐101).

### 2.2 Primary mouse hepatocyte isolation

Primary mouse hepatocytes were isolated from 9‐week‐old mice fed an NC diet ad libitum, as previously reported, with slight modifications (Charni‐Natan & Goldstein, 2020). The details of the solutions used for liver perfusion are presented in Table S2. Mice were anesthetized using 0.015 mg/kg BW Dorbene‐Vet (Kyoritsu Seiyaku, Tokyo, Japan), 77 0.75 mg/kg BW Dormicum (Astellas Pharma, Tokyo, Japan), and 0.75 mg/kg BW Vetorphale (Meiji Pharma, Tokyo, Japan). Anesthesia was conducted by intraperitoneal injection. Briefly, the portal vein was cannulated and perfused in situ with EGTA solution (pH 7.5, 2.5 mL/min, 37°C for 4 min), followed by collagenase solution (C0130, Sigma Aldrich, Tokyo, Japan) (pH 7.5, 2.5 mL/min, 37°C for 4 min). The liver was removed and gently dissolved in a fresh collagenase solution. The digest was filtered through a cell strainer (100 *μ*m) with 40 mL Hanks' solution (pH 7.5, 4°C) and centrifuged at 50 × *g* for 3 min, 150 × *g* for 7 min, and 50 × *g* for 3 min. Primary hepatocytes were seeded at 2 × 10^5^ cells/well in a collagen‐coated 24‐well plate (4820‐010, AGC Techno Glass, Shizuoka, Japan), in Williams' E Medium (W4128, Sigma Aldrich) containing 10% fetal bovine serum (A5256701, Gibco Life Science, Tokyo, Japan), 1 *μ*M ITS‐A (097‐06751, Fujifilm, Osaka, Japan), 1 *μ*M dexamethasone (047‐18863, Fujifilm), and incubated at 37°C in a 5% CO_2_ atmosphere. After an initial 3‐h incubation, the medium was replaced with fresh medium and incubated for 24‐h.

### 2.3 Screening of FGF21‐inducers

A total of 46 rare sugars were screened (Table S3). Following the initial incubation, primary hepatocytes were treated with culture medium containing vehicle (distilled water) or 25 mM sugar for 24 h. We chose this concentration of the sugars based on the previous report (Iroz et al., 2017), where significant induction of *Fgf21* mRNA was observed in mice primary hepatocyte culture by D‐glucose. The medium was collected and FGF21 levels were measured using a Mouse/Rat FGF‐21 Quantikine ELISA Kit (MF2100, R&D Systems, USA) according to the manufacturer's instructions.

### 2.4 Plasma FGF21 measurement

Nine‐week‐old mice received a gastric gavage of the vehicle (distilled water) or FGF21‐inducing rare sugars from the screening step at a dose of 5 g/kg body weight (BW), equivalent to 12.4 mL/kg of 0.4 g/mL solution. The gavage volume was approximately 0.26 mL per mouse and delivered slowly over approximately 1 s, to minimize stress and avoid regurgitation. Blood samples were collected from the tail vein at 0 h (baseline) and 4, 6, 8, 10, 12, and 24 h after gavage. Mice were fed ad libitum before and after the administration. Plasma samples were prepared from the blood samples and stored at −80°C until use. Mouse FGF21 protein levels in each plasma sample were determined using a Mouse/Rat FGF‐21 Quantikine ELISA Kit (R&D Systems) according to the manufacturer's instructions.

### 2.5 Immunohistochemistry of PVH^Oxt^

c‐Fos and PVH^Oxt^ immunohistological procedures were performed according to a previous report, with adjustments (Perrin‐Terrin et al., 2016). NC‐fed 9‐week‐old mice were fasted for 2 h prior to induction. Gastric gavage was performed with either vehicle (distilled water) or FGF21‐inducing rare sugars at 5 g/kg body weight and left in the light cycle for 6 h with free access to water until brain harvest. Mice were anesthetized using 0.015 mg/kg BW Dorbene‐Vet (Kyoritsu Seiyaku, Tokyo, Japan), 0.75 mg/kg BW Dormicum (Astellas Pharma, Tokyo, Japan), and 0.75 mg/kg BW Vetorphale (Meiji Pharma, Tokyo, Japan). Anesthesia was conducted by intraperitoneal injection. Mice were perfused transcardially with phosphate‐buffered saline (PBS) (166‐23555, Fujifilm) followed by 4% paraformaldehyde (PFA) (162‐16065, Fujifilm) for 5 min each. The brain was harvested, submerged in fresh PFA solution, and fixed overnight at 4°C. Following overnight fixation, the brain was transferred to a 30% sucrose solution and kept for 24 h or until use at 4°C. The coronal section of the brain was prepared at 30 μm in thickness and kept in a cryoprotectant (50% [vol. /vol.] PBS, 30% [vol./vol.] mL of ethylene glycol, and 20% [vol. /vol.] glycerol).

The brain slices were sequentially washed three times in PBS, followed by 15 min of incubation in PBS containing 0.3% H_2_O_2_. Subsequent washing and blocking were performed (PBS, 10% normal donkey serum (IHR‐8135, Funakoshi, Tokyo, Japan), and 0.1% Triton X‐100 (12968‐35, Nacalai Tesque, Inc., Kyoto, Japan)) for 40 min. c‐Fos primary antibody (ABE457, Merck Millipore, Tokyo, Japan) was added and incubated at room temperature overnight. The slices were washed, incubated with secondary antibody (K500711‐2, Dako REAL EnVision Detection System, Agilent, Santa Clara, USA), and visualized according to the manufacturer's protocol. PVH^Oxt^ staining was performed following c‐Fos visualization in the same sequence without nickel addition for the visualization. Brain slices were air‐dried overnight at room temperature and encapsulated in Eukitt (6.00.01.0001.06.01.EN, ORSAtec, Bobingen, Germany) after dehydration in absolute ethanol and xylene.

### 2.6 Gastric gavage two‐bottle choice test

Eight‐ to nine‐week‐old sugar‐naïve mice were individually housed in sterilized cages with free access to the NC diet and two bottles containing either vehicle (distilled water) or 50 mM sucrose solution for 3 days. Gastric gavages, as described in Section 2.4, were given in the following experimental order: vehicle (distilled water) for 2 days (Days 1 and 2), followed by either vehicle or 5 g/kg BW FGF21‐inducing rare sugars for 2 days (Days 3 and 4). Mice in each group received only one type of solution on Days 3 and 4, either vehicle or one of the FGF21‐inducing rare sugars. The gavages were given once a day in the light phase, approximately 6 h prior to the dark phase. Two bottles containing either vehicle (distilled water) or 50 mM sucrose solution were given ad libitum, following the gavage. The position of the bottles was switched daily to prevent positional bias. Solution intake was measured daily, and sucrose preference was expressed as the percentage of sucrose consumption over total liquid consumption. Data for “pre” and “post” were obtained on Days 1 and 2, or Days 3 and 4, respectively.

### 2.7 Ad libitum two‐bottle choice test

Eight‐ to nine‐week‐old sugar‐naïve mice were individually housed in sterilized cages with free access to NC diet and two bottles containing either vehicle (distilled water) or 100 mM sucrose solution for 3 days. Subsequently, we gave two choices of solutions in the following order: vehicle (distilled water) versus 100 mM sucrose for 2 days, followed by vehicle (distilled water) versus 100 mM sucrose with 600 mM D‐glucose (as the positive control for sweet taste) for 2 days and ended with vehicle (distilled water) versus 100 mM sucrose with 600 mM of one of the FGF21‐inducing rare sugars for 2 days. Mice were left to feed and drink ad libitum over a period of 2 days for each choice. Each mouse was assigned to only one experimental group, and only one rare sugar was tested in each mouse. Daily solution intake was recorded, and sucrose preference was expressed as the percentage of sucrose consumption over total liquid consumption.

### 2.8 Long‐term lick analysis

The lick analytic choice preference experiment system (LKP2, Melquest corporation, Toyama, Japan) was used in this analysis. Mice were trained and tested in licking test chambers (LKP2‐MC, interior dimensions: 165 × 250 × 190 mm). Solutions were delivered through drinking spouts attached to 15‐mL glass tubes. Licking test chambers were connected to a contact lick interface (*LKP‐IF*), which transmitted licking frequency (per second) to a computer. The software (*LKP2‐AN*) was used to analyze the data transmitted from the interface. Recorded data included burst number, mean burst size, and mean inter‐burst intervals. Data were collected in MS Excel 2020, while an interbout interval of 1000 ms was used (a duration of 1000 ms or longer between two licks defined the end of one lick and the start of the next). In other words, lick bursts were defined as any sequence of licks separated by <1 s, and the end of a burst was marked by a pause of at least 1 s.

The mice were first acclimatized to *LKP2‐MC* with two bottles that contained water for 4 days. Subsequently, they were provided ad libitum access to two bottles containing vehicle (distilled water) or test solution. 100 mM sucrose solution was presented as the test solution for 2 days, followed by the test of the mixture of 100 mM sucrose plus 600 mM D‐allulose (final concentrations) for another 2 days in *LKP2‐MC*. Therefore, mice spent a total of 8 consecutive days in the *LKP2‐MC* cage. The position of the bottles was switched daily to exclude position bias. The licking patterns of the test solutions during the dark cycle over 2 days of testing were analyzed using *LKP2*.

### 2.9 Data visualization and statistical analysis

Analyses were done using MS Excel 2020 and GraphPad Prism 10. Data are expressed either as mean ± standard deviation (SD) or box‐whiskers, where the middle line represents the median, bottom/top edges represent the 25th/75th percentile of the data, and whiskers represent the maximum and minimum value of the data. Statistical analysis between two groups was performed using Student's paired *t‐*test, one‐way ANOVA, and repeated‐measures ANOVA, followed by Dunnett's or Tukey's HSD post hoc test. Statistical significance was set at *p* < 0.05.

## 3 RESULTS

### 3.1 Identification of FGF21‐inducing rare sugars

**FIGURE 1:**
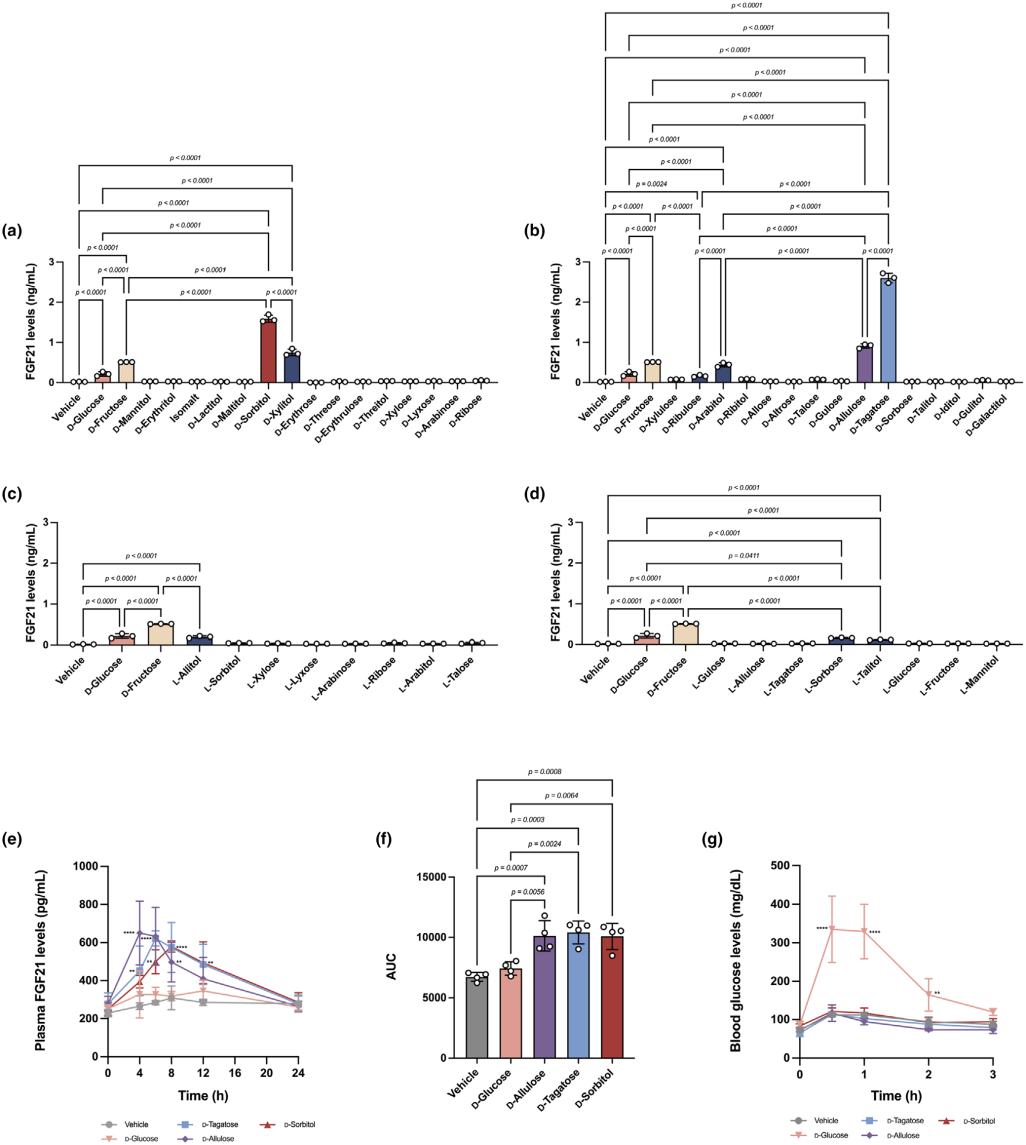
Identification of FGF21‐inducing rare sugars and their effects on blood glucose. (a–d) FGF21 level of mouse primary hepatocytes treated for 24 h with vehicle (distilled water‐negative control) or 25 mM D‐glucose, D‐fructose (positive control), or rare sugars (46 samples). *n* = 3 mice. (e) Plasma FGF21 levels over 24 h after gastric gavage of vehicle (distilled water), D‐glucose, D‐tagatose, D‐allulose, or D‐sorbitol at 5 g/kg in nine‐week‐old male WT mice with ad libitum access to normal chow (NC) diet. *n* = 4 mice per group. (f) Area under the curve (AUC) over 24 h of data in (e). *n* = 4 mice per group. (g) Blood glucose levels of the mice receiving the same dose in (e) of either vehicle (distilled water), D‐glucose, D‐tagatose, D‐allulose, or D‐sorbitol after 16 h fasting. *n* = 4 mice per group. Statistical analyses were done by one‐way ANOVA for (a–d, f) and repeated measures ANOVA for (e, g), followed by Tukey's HSD test. **p < 0.01; ****p < 0.0001.

In order to identify FGF21‐inducing sugars, 46 rare sugars were assayed by in vitro screening using mouse primary hepatocytes because primary cells maintain endogenous patterns of lipid and glucose metabolism better than cell lines (Nagarajan et al., 2019). Nine rare sugars (D‐allulose, D‐tagatose, D‐sorbitol, D‐xylitol, D‐ribulose, D‐arabitol, L‐allitol, L‐sorbose, and L‐talitol) significantly induced Fgf21 secretion when compared to vehicle (distilled water) (Figure 1a–d), while only four of these (D‐allulose, D‐tagatose, D‐sorbitol, and D‐xylitol) were significant compared to positive controls (D‐glucose and D‐fructose) (Figure 1a,b). D‐allulose, D‐tagatose, and D‐sorbitol have not been reported as FGF21 inducers, whereas D‐xylitol has been previously reported (Uebanso et al., 2017).

We tested the FGF21‐inducing capacity of D‐allulose, D‐tagatose, and D‐sorbitol in vivo in BL/6 mice by serially measuring plasma FGF21 and blood glucose levels after administration of 5 g/kg BW of each sugar by gastric gavage. The peak level and timing of plasma FGF21 differed among the inducers (Figure 1e,f), but they significantly increased the plasma FGF21 levels compared to D‐glucose. In contrast, these sugars had minimal effects on blood glucose levels (Figure 1g), indicating that they induced FGF21 secretion without affecting blood glucose levels in mice.

### 3.2 FGF21‐inducing rare sugars activated PVH^Oxt^

**FIGURE 2:**
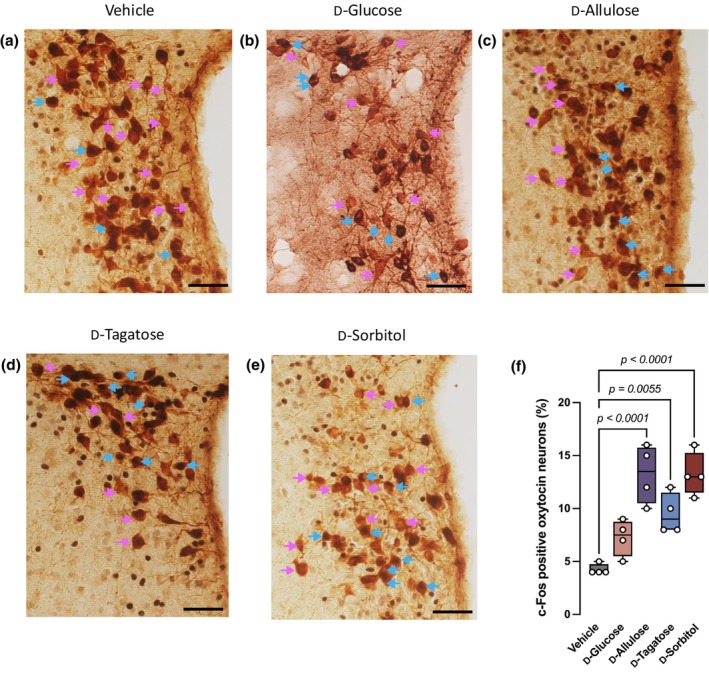
FGF21‐inducing rare sugars activated oxytocin neurons of paraventricular nucleus of hypothalamus in BL/6 mice. (a–e) Immunostaining of mice brain after gastric gavage with either (a) vehicle (distilled water) or 5 g/kg body weight of D‐glucose (b), D‐tagatose (c), D‐allulose (d), or D‐sorbitol (e). Blue arrow (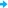) indicates c‐Fos‐positive oxytocin neurons, shown by dark brown spot on the light brown neurons (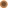). Magenta arrow (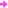) indicates c‐Fos‐negative oxytocin neurons, shown by light brown anti‐oxytocin staining (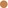). *n* = 4 mice per group. Scale bar represents 100 *μ*m. (f) Percentage of activated oxytocin neurons (c‐Fos positive oxytocin neurons) from the total of PVH oxytocin neurons in the mice receiving either vehicle (distilled water), D‐glucose, or FGF21‐inducing rare sugars in (a–e). Data are presented as box‐whiskers in (f), where the middle line represents median, bottom/ top edges represent 25th/75th percentile of the data, and whiskers represent maximum and minimum value of the data. Statistical analyses were done by one‐way ANOVA, followed by Dunnett's test.

FGF21‐PVH^Oxt^ post‐ingestive signal is activated by the ingestion of dietary sugars, and approximately 12% of PVH^Oxt^ neurons are activated by the IP administration of recombinant FGF21 to mice (Matsui et al., 2018). To test the efficacy of FGF21‐inducing rare sugars in vivo, we used the activation of PVH^Oxt^ neurons as a surrogate marker for the potency of FGF21‐inducing rare sugars and compared them with D‐glucose. The brains of mice that received a gastric gavage of 5 g/kg BW of the inducers were analyzed histologically using the neuronal activation marker c‐Fos. There was a significant activation of PVH^Oxt^ neurons in mice receiving FGF21‐inducing rare sugars compared to the vehicle (distilled water) (Figure 2a–f). In contrast, gastric gavage of D‐glucose only modestly increased the number of c‐Fos positive PVH^Oxt^ neurons. Therefore, D‐allulose, D‐tagatose, and D‐sorbitol induce FGF21 secretion in vivo and subsequently activate PVH^Oxt^ neurons, and they are more potent than D‐glucose.

### 3.3 FGF21‐inducing rare sugars reduced sweet intake and preference in mice

In order to evaluate the effects of these FGF21‐inducing sugars on sugar appetite, we performed two tests. Appetite regulation is influenced by both oral and post‐oral mechanisms, both of which include taste receptors and endocrine signaling (Chaudhari & Roper, 2010; Sclafani & Ackroff, 2012). Gastric gavage bypasses oral signaling by the taste receptors. In contrast, providing rare sugars by mixing in solution stimulates both oral and post‐oral mechanisms.

**FIGURE 3:**
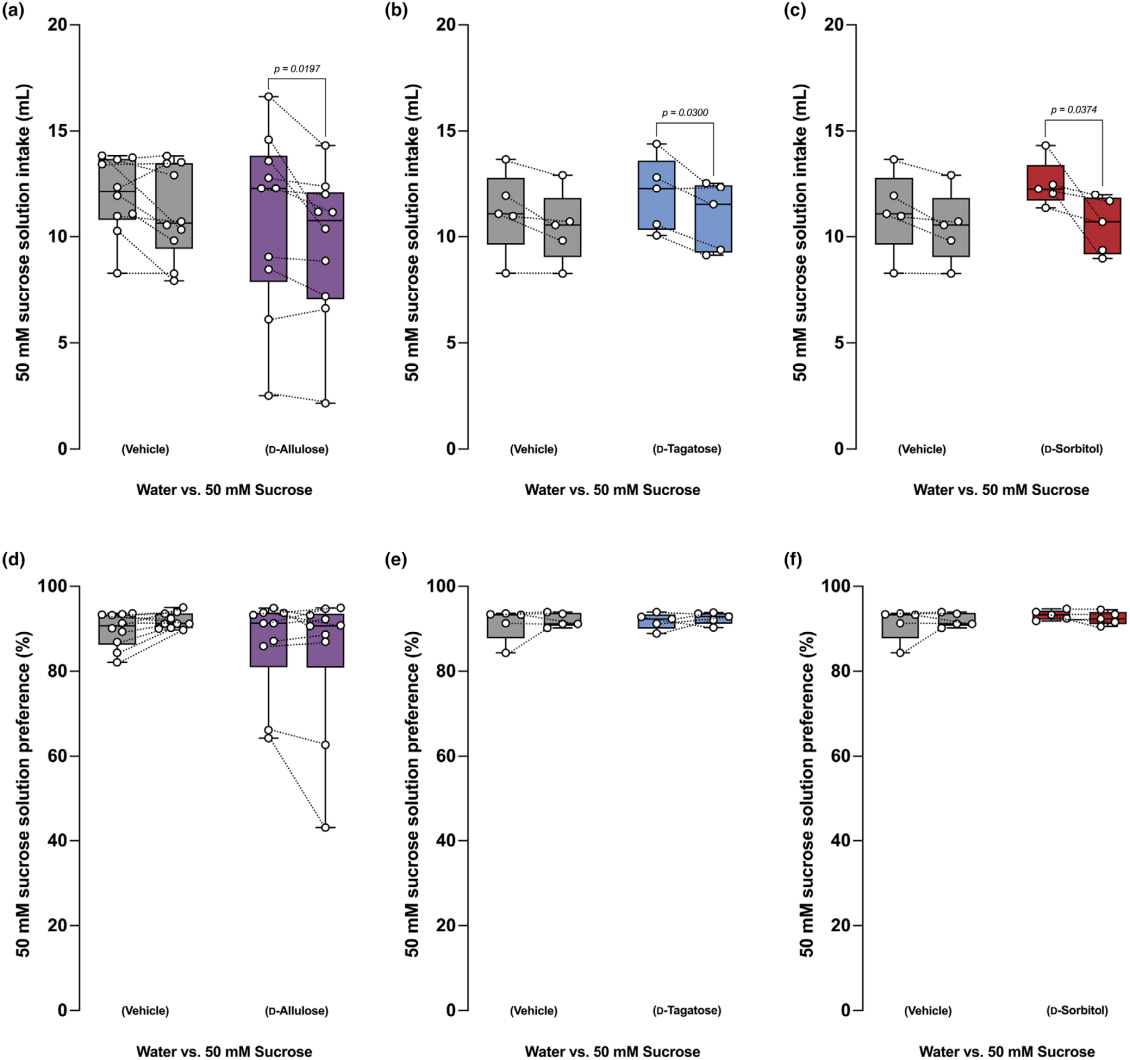
Intragastric administration of FGF21‐inducing rare sugars reduced sucrose preference in BL/6 mice. (a–c) 50 mM sucrose intake in mice receiving 5 g/kg body weight of D‐allulose (a), D‐tagatose (b), or D‐sorbitol (c). (d–f) sucrose preference of the same mice in (a–c). *n* = 10 mice per group for (a, d) and *n* = 5 mice per group for (b, c, e, f). Data are presented as box‐whiskers, where the middle line represents the median, bottom/top edges represent the 25th/75th percentile of the data, and whiskers represent the maximum and minimum value of the data. Statistical analyses were done by Student's paired *t*‐test between “pre” and “post” of each treatment.

We first administered rare sugars to mice by gastric gavage at 5 g/kg BW and evaluated their sugar‐ingestive behavior using a two‐bottle choice test between water and 50 mM sucrose for 2 days. Gastric administration of these sugars compared to distilled water significantly reduced the intake of 50 mM sucrose solution in mice (Figure 3a–c), but it was not sufficient to reduce sucrose preference (Figure 3d–f).

**FIGURE 4:**
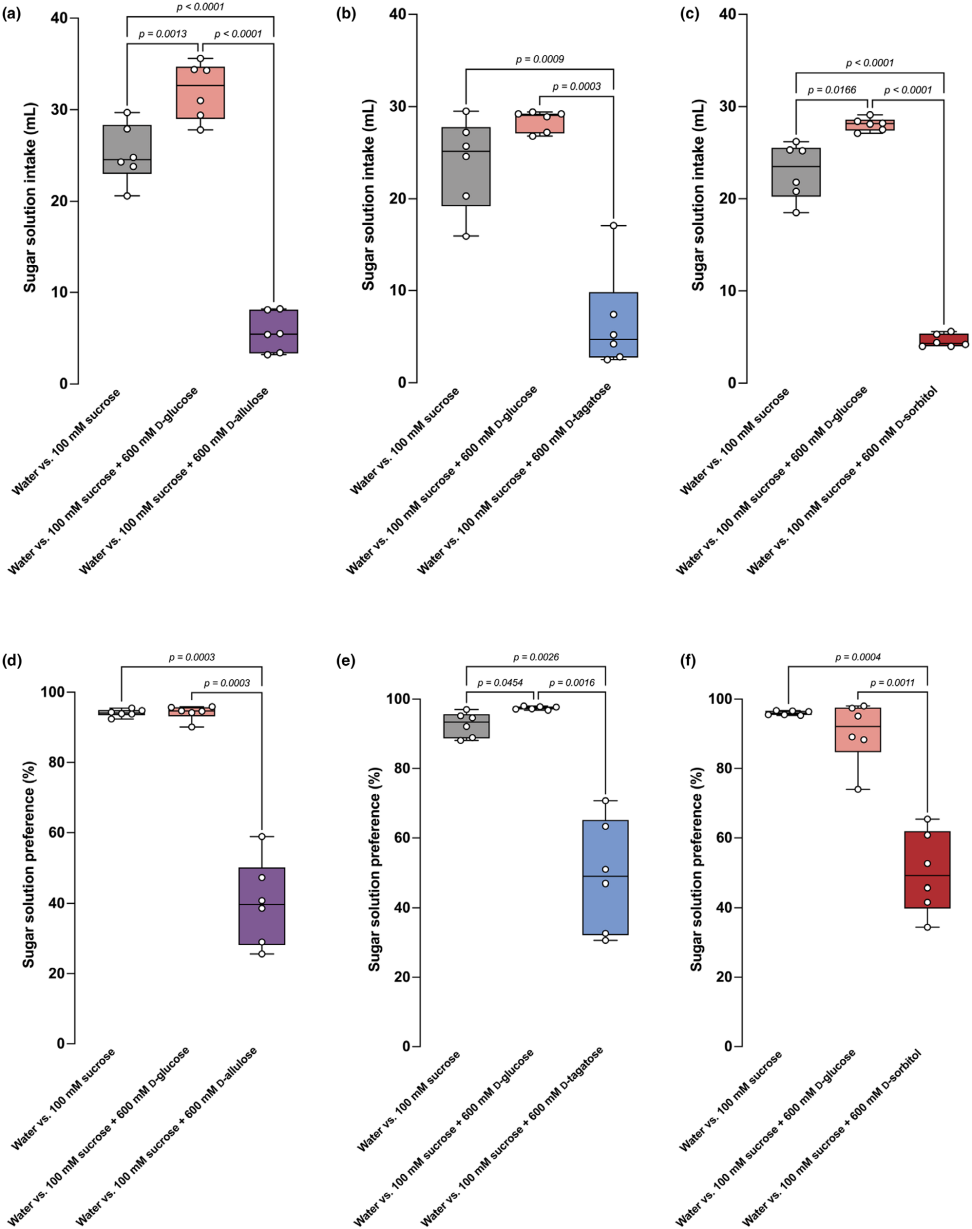
Mixing FGF21‐inducing rare sugars into sucrose solution reduced solution intake and preference in BL/6 mice. (a–c) Intake of 100 mM (final concentration) sucrose solution that was mixed with either vehicle (distilled water), 600 mM (final concentration) of D‐glucose, or 600 mM (final concentration) of D‐allulose (a), D‐tagatose (b), or D‐sorbitol (c). (d–f) Sucrose preference of the same mice in (a–c). *n* = 6 mice per group. Data are presented as box‐whiskers, where the middle line represents the median, bottom/top edges represent the 25th/75th percentile of the data, and whiskers represent the maximum and minimum value of the data. Statistical analyses were done by repeated measures ANOVA, followed by Tukey's HSD test.

Next, to avoid the possibility of the daily gavage procedure, which may be stressful, affecting the ingestive behavior of mice, we mixed rare sugars one at a time into a sucrose solution and let mice consume the solution ad libitum. We increased the concentration of the sucrose solution from 50 mM to 100 mM, hoping to promote voluntary intake of the solution with rare sugars. In this experimental design, the consumption of the sucrose solution in the vehicle (water)‐mixed solution group increased compared to the vehicle (water)‐gavaged group (Figures 3a–c and 4a–c). In this mixture experiment, we used D‐glucose as a sweet taste control sugar that has a similar sweetness but weaker FGF21‐inducing capacity compared to the identified FGF21‐inducing rare sugars. Adding D‐glucose to the final concentration of 600 mM in a 100 mM sucrose solution increased solution intake by mice. In contrast, adding each FGF21‐inducing sugar to the final concentration of 600 mM in a 100 mM sucrose solution significantly reduced the solution intake (Figure 4a–c) and preference (Figure 4d–f).

Therefore, these sugars showed stronger effects in reducing sucrose solution intake when given by mixture than by once‐a‐day gavage. The stronger effect seen by the mixture solution may be due to the addition of taste effects, longer and more frequent exposure (once‐a‐day dosing vs. exposure during each ingestion), and/or a less stressful method of administration (daily forced gavage vs. voluntary intake).

### 3.4 Appetitive drive for sucrose is suppressed by D‐allulose

**FIGURE 5:**
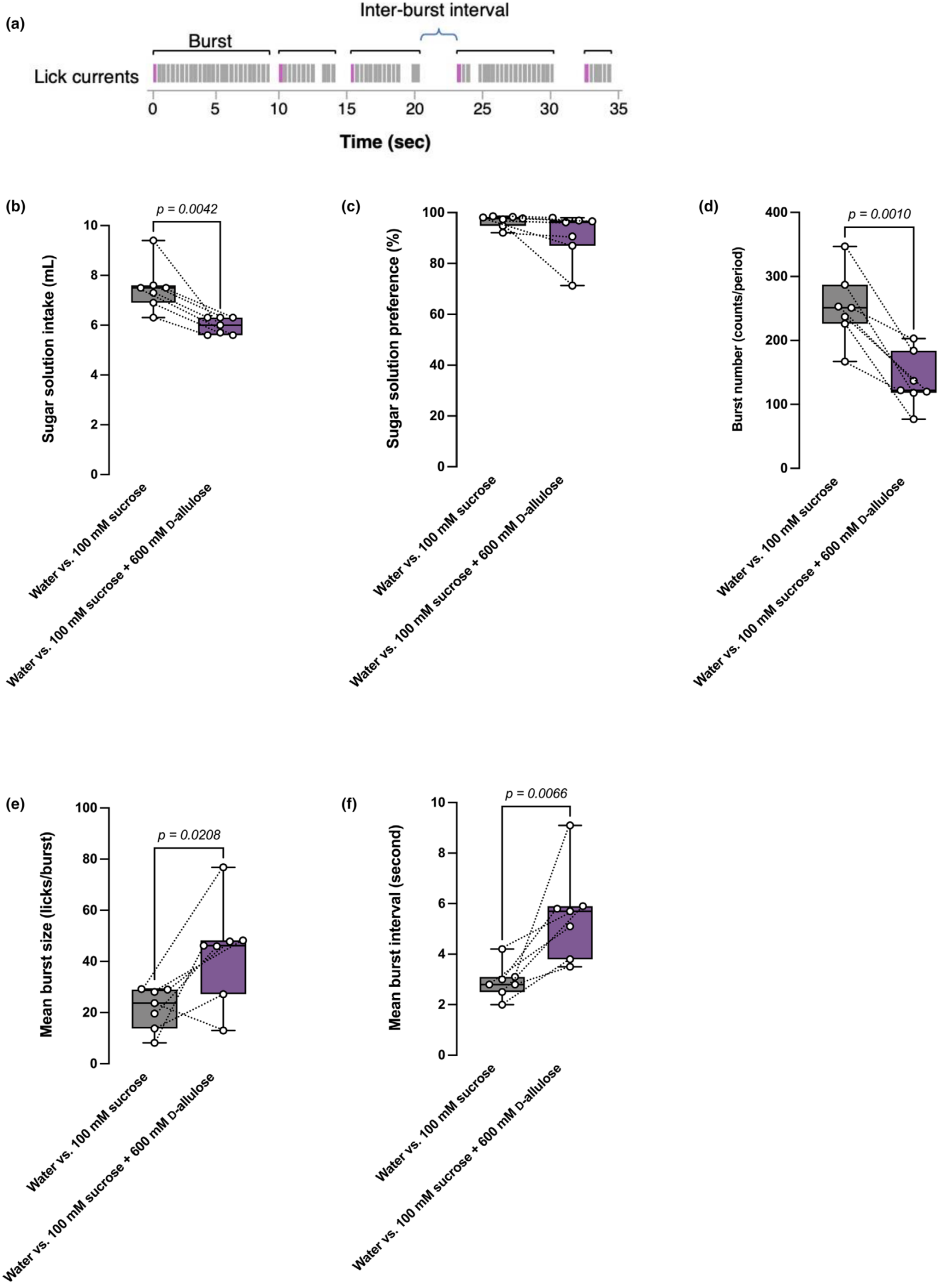
D‐allulose reduced the appetitive drive for sucrose and prolonged the inter‐drink interval. (a) Lick microstructure illustration. (b, c) Intake (b) of and preference (c) for 100 mM sucrose solution containing vehicle (distilled water) or 600 mM D‐allulose for 2 days in mice. *n* = 7 mice per group. (d–f) Lick indices for 100 mM (final concentration) sucrose with/without 600 mM (final concentration) D‐allulose versus water over two dark cycles in mice. Mean burst size (d), burst number (e), and mean inter‐burst interval (f). *n* = 7 mice per group. Data are presented as box‐whiskers, where the middle line represents the median, bottom/top edges represent the 25th/75th percentile of the data, and whiskers represent the maximum and minimum values of the data. Statistical analyses were done by Student's paired *t‐*test.

To dissect the effect of these sugars on reducing sucrose intake, we used a lick apparatus, which measured the licking pattern and frequency of certain solutions by test subjects. The appetite for and palatability of a solution can be distinguished by analyzing clusters of licking called bursts (Fushimi et al., 2025; Naneix et al., 2019). Burst size, burst number, and interburst interval measured using liquid would correspond to meal size, meal frequency, and intermeal interval measured using food, respectively. Mean burst size reflects the effects of orosensory stimuli and is associated with palatability, whereas the number of bursts (burst number) is influenced by post‐ingestive consequences and is associated with the desire state, namely appetite. The interburst interval represents the duration of the replete state, which is affected by the need state of the subject (Figure 5a). We devised the lick analyses so that we can assess the influence of post‐ingestive negative‐feedback effects on ingestive behavior. Therefore, we extended the duration of our observation to 12 h and assessed the effect of between‐drink events on within‐drink events.

We analyzed D‐allulose, which showed the strongest effects among three FGF21‐inducing sugars identified. We also evaluated the effect of mixing D‐allulose with a sucrose solution by performing 12 h lick analyses with two bottle choices: water and the test solution. This experimental setting allowed us to measure appetite, palatability, and inter‐drink intervals, along with the intake of and preference for the solutions. The addition of D‐allulose significantly reduced sucrose intake in the mice (Figure 5b), but not the preference for the sucrose solution (Figure 5c). Mean burst size increased significantly. In contrast, the burst number was significantly reduced (Figure 5e), and the interburst interval was significantly longer (Figure 5f). These data suggest that D‐allulose can suppress appetite despite increasing palatability and extend the replete state, resulting in a reduced total intake of sucrose‐containing solutions.

## 4 DISCUSSION

Our study has revealed that D‐tagatose, D‐allulose, and D‐sorbitol are FGF21‐inducers and that the administration of these sugars elevates plasma FGF21 levels and subsequently activates PVH^Oxt^ neurons in mice. When FGF21‐inducing rare sugars were administered directly into the stomach, they reduced sucrose intake but not sucrose preference. In contrast, when one of these sugars was mixed with a sucrose solution, each of them significantly reduced both the intake and preference. It contrasts with D‐glucose, which has a similar sweetness to these sugars but is less capable of raising plasma FGF21 concentrations. Continuous supply of these sugars at each drink event through mixed solution than giving them by once‐a‐day gavage may help to sustain the effect and have more impact on reducing cumulative intake over time. In various clinical trials, D‐allulose has been used at doses of 5–30 g/day for various durations without significant side effects (Ahmed et al., 2021; Franchi et al., 2021; Tanaka et al., 2020). However, its mechanism of action remains unclear. To the best of our knowledge, this is the first report on rare sugars as FGF21 inducers and post‐ingestive FGF21‐PVH^Oxt^ signal activators, which reduce sugar appetite.

Although the oral dose at 5 g/kg BW of mice was higher compared to the dose used in a previous report (Iwasaki et al., 2018), we did not observe any diarrhea in our mice. The human equivalent dose (HED) for 5 g/kg BW of rare sugar in mice is 0.4 g/kg BW (Nair & Jacob, 2016). For an adult with 60 kg of body weight, it corresponds to 24 g of rare sugars. Previous clinical trials using rare sugar at doses ranging between 5 and 30 g/day were conducted without any side effects (Ahmed et al., 2021). D‐allulose and D‐tagatose are classified as “generally regarded as safe” (GRAS) by the FDA and pose no safety risk (GRN 352; GRN 693) (US Food and Drug Administration, n.d.a, n.d.b). However, cautions are needed as previous reports have shown the incidence of gastrointestinal symptoms when higher amounts of D‐allulose and D‐tagatose were ingested (Ensor et al., 2014; Han et al., 2018).

We observed the reduction in the preference for the D‐allulose‐mixed sucrose solution in the two‐bottle‐choice test (Figure 4d), but not in the lick analysis (Figure 5c). There were several differences in the experimental conditions between these two experiments. The former was conducted in the home cages whereas the latter was conducted in the lick cages. In the ad libitum two‐bottle‐choice test, mice experienced the D‐glucose‐mixed sucrose solution prior to the D‐allulose‐mixed sucrose solution. The D‐glucose‐mixed sucrose solution contains four times more calories than the D‐allulose‐mixed sucrose solution. The mice in the two‐bottle‐choice test exposed longer to a solution with higher energy may have acquired a learned satiety (Yeomans et al., 2005) to sweet tastant. Therefore, the difference in cages, length of exposure to sweet tastant, and caloric density of the solution used may have generated the difference in Figures 4d and 5c.

The differences in the degree of sugar appetite suppression among FGF21‐inducing C6 sugars, while not statistically significant, may be attributed to the different properties of these sugars and their metabolism in the body. The absorption and metabolism of D‐allulose and D‐tagatose, the epimers of fructose, and sorbitol, a sugar alcohol, are different. Orally ingested D‐allulose is absorbed from the intestine, accumulates in the liver, remains mostly unmetabolized, and is excreted from the kidneys (Maeng et al., 2019; Tsukamoto et al., 2014). D‐tagatose is poorly absorbed and metabolized, once absorbed at 13%–39% of the initial oral intake (Saunders et al., 1999); thus, less tagatose may reach the liver compared to D‐allulose, which may explain its weaker sweet intake suppression compared to D‐allulose in vivo, despite being a stronger FGF21‐inducer in vitro.

While D‐allulose, D‐tagatose, and D‐sorbitol induced FGF21 secretion and activated PVH^Oxt^, whether these effects are required for reducing sugar appetite remains elusive. We also observed c‐Fos‐positive/Oxt‐negative neurons in the PVH sections, suggesting that other neuronal populations in the PVH activated by these sugars might also contribute to the effect of these sugars to reduce sugar intake. On the other hand, sugar intake is regulated by various factors, including the gut‐brain axis. D‐allulose elevates GLP‐1 production and subsequently suppresses feeding (Iwasaki et al., 2018), which may serve as an alternative post‐ingestive signal for controlling sugar intake. A limitation of the current study was that we could not clarify the degree of contribution of various effectors of FGF21‐inducing rare sugars in reducing sugar appetite.

## 5 CONCLUSION

In conclusion, D‐allulose, D‐tagatose, and D‐sorbitol induce FGF21 secretion, activate oxytocin neurons in the paraventricular nucleus of the hypothalamus, and reduce sugar appetite. FGF21‐inducing sugars are affordable and are Generally Recognized as Safe food additives. This could serve as a more feasible measure for controlling sugar intake.

## AUTHOR CONTRIBUTIONS

OGHNBE was involved in conceptualization, methodology, investigation, formal analysis, and writing—original draft. SM was involved in conceptualization, methodology, investigation, formal analysis, supervision, and writing—review and editing. ST was involved in writing—review and editing. TS was involved in conceptualization, methodology, supervision, and writing—review and editing.

## FUNDING INFORMATION

This study was supported by the following grants to T.S.: JSPS KAKENHI (grant number: 20H00412) and grants from the Japan Foundation for Applied Enzymology, Naito Foundation, Kao Health Science Foundation, Sumitomo Foundation, MSD Life Science Foundation, Uehara Memorial Foundation, and the Foundation for Dietary Scientific Research. This study was supported by the following grants to S.M.: grants from the Lotte Foundation, TOBE MAKI Scholarship Foundation, Kowa Life Science Foundation, Japan Food Chemical Research Foundation, Kyoto University Foundation, and ISHIZUE 2022 of Kyoto University. We are grateful to the Ministry of Education, Culture, Sports, Science, and Technology of Japan and the Foundation for Dietary Scientific Research for providing scholarships to support OGHNBE.

## CONFLICT OF INTEREST STATEMENT

Sho Matsui and Tsutomu Sasaki are inventors of the patent (application number 2021‐574140 (JP); international patent application number PCT/JP2021/3182; international patent publication number WO2021/153718) entitled “FIBROBLAST GROWTH FACTOR 21 INDUCER, AND COMPOSITION FOR SUPPRESSING ALCOHOL PREFERENCE OR SIMPLE SUGAR PREFERENCE.” The other authors declare no competing financial or nonfinancial interests.

## ETHICS STATEMENT

All animal experiments were conducted in accordance with the Regulations of Animal Experimentation of Kyoto University (approval number: 31‐75 and R2‐101).

## Supporting information


Tables S1–S3.

## Data Availability

All data in this study is available upon request.
