# Correction to “Identification of FGF21‐inducing rare sugars that reduces sugar appetite in male BL/6 mice”

**DOI:** 10.14814/phy2.70678

**Published:** 2026-04-17

Oulan G. H. N. B. Efendi, Sho Matsui, Satoshi Tsuzuki, Tsutomu Sasaki. Identification of FGF21‐inducing rare sugars that reduces sugar appetite in male BL/6 mice. *Physiol Rep*. First published: 15 October 2025, https://doi.org/10.14814/phy2.70618.

The authors regret several text errors for numerical values and referencing of figure panels in the published paper.

In “1. INTRODUCTION”, the 2nd last sentence “We have hypothesized that this reduction in food intake may be mediated through the activation of FGF21 the PVH ^Oxt^” was incorrect. This should have read: “We have hypothesized that this reduction in food intake may be mediated through the activation of **FGF21‐PVH** ^**Oxt**^ **post‐ingestive signal**.”

In “2. MATERIALS AND METHODS, 2.2 Primary mouse hepatocyte isolation”, the 3rd sentence “Mice were anesthetized using 0.015 mg/kg BW Dorbene‐Vet (Kyoritsu Seiyaku, Tokyo, Japan), 77 0.75 mg/kg BW Dormicum (Astellas Pharma, Tokyo, Japan), and 0.75 mg/kg BW Vetorphale (Meiji Pharma, Tokyo, Japan)” was incorrect. This should have read: “Mice were anesthetized using 0.015 mg/kg BW Dorbene‐Vet (Kyoritsu Seiyaku, Tokyo, Japan), **0.75 mg/kg BW** Dormicum (Astellas Pharma, Tokyo, Japan), and 0.75 mg/kg BW Vetorphale (Meiji Pharma, Tokyo, Japan).”

In “2 MATERIALS AND METHODS, 2.8 Long‐term lick analysis”, there were several mistakes. Sentences “100 mM sucrose solution was presented as the test solution for 2 days, followed by the test of the mixture of 100 mM sucrose plus 600 mM D‐allulose (final concentrations) for another 2 days in LKP2‐MC. Therefore, mice spent a total of 8 consecutive days in the LKP2‐MC cage. The position of the bottles was switched daily to exclude position bias. The licking patterns of the test solutions during the dark cycle over 2 days of testing were analyzed using LKP2.” were incorrect. These should have read: “100 mM sucrose solution was presented as the test solution for **1 day**, followed by the test of the mixture of 100 mM sucrose plus 600 mM D‐allulose (final concentrations) for another **1 day** in LKP2‐MC. Therefore, mice spent a total of **6** consecutive days in the LKP2‐MC cage. The position of the bottles was switched daily to exclude position bias. The licking patterns of the test solutions during the dark cycle over **1 day** of testing were analyzed using LKP2.”

In the Figure 2 legend, the description “(a–e) Immunostaining of mice brain after gastric gavage with either (a) vehicle (distilled water) or 5 g/kg body weight of D‐glucose (b), D‐tagatose (c), D‐allulose (d), or D‐sorbitol (e)” was incorrect. This should have read: “(a–e) Immunostaining of mice brain after gastric gavage with either (a) vehicle (distilled water) or 5 g/kg body weight of D‐glucose (b), **D‐allulose (c), D‐tagatose (d)**, or D‐sorbitol (e).”

In “Figure 5 (f ) Mean burst interval”, the unit of the Y axis “(second)” was incorrect. The unit should have been: “(**minute**)”.

In the “Figure 5 legend,” the sentence “Mean burst size (d), burst number (e), and mean inter‐burst interval (f)” was incorrect. This should have read: “**Burst number (d), mean burst size (e)**, and mean inter‐burst interval (f).”

In Section 3. RESULTS—“3.4 Appetitive drive for sucrose is suppressed by D‐allulose,” the sentence “In contrast, the burst number was significantly reduced (Figure 5e), and the interburst interval was significantly longer (Figure 5f)” was incorrect. This should have read: “In contrast, the burst number was significantly reduced (**Figure 5d**), and the interburst interval was significantly longer (Figure 5f).”

We apologize for these errors, which do not affect the scientific conclusions of the study.

