# Supplementary material for: Identification of FGF21‐inducing rare sugars that reduces sugar appetite in male BL/6 mice
Source: Physiol Rep. 2025 Oct 15;13(20):e70618. doi: 10.14814/phy2.70618 (PMC12521948; doi:10.14814/phy2.70618)
Supplement: Supplementary file 1 — Tables S1–S3. [file PHY2-13-e70618-s001.docx]

**Supporting Information**

**Supplementary Table 1.** Nutritional composition of the normal chow (NC) diet

| **Component** | **(%)/100 g** | **kcal/100 g** |
| --- | --- | --- |
| Protein | 25.06 | 100.2 |
| Fat | 4.78 | 43.02 |
| Fiber | 4.91 | - |
| Ash | 6.88 | - |
| Moisture | 7.95 | - |
| NfE | 50.42 | 201.7 |
| **Total** |  | **344.92** |

**Supplementary Table 2.** Composition of solutions used for primary hepatocyte isolation (1 liter solution).

*Hank’s Solution*

| **Component** | **SOURCE** | **IDENTIFIER** | **CAS** | **CONCENTRATION (mM)** |
| --- | --- | --- | --- | --- |
| NaCl | FUJIFILM Wako | 191-01665 | 7647-14-5 | 136.9 |
| KCl | Nacalai Tesque | 28514-75 | 7447-40-7 | 5.4 |
| Na_2_HPO_4_.12H_2_O | FUJIFILM Wako | 193-02845 | 10039-32-4 | 0.3 |
| KH_2_PO_4_ | Nacalai Tesque | 2871-55 | 7778-77-0 | 0.4 |
| MgSO_4_.7H_2_O | Wako Pure Chemical Industries, Ltd. | 131-00405 | 10034-99-8 | 0.8 |
| CaCl_2_ | Nacalai Tesque | 06729-55 | 10043-52-4 | 1.8 |
| NaHCO_3_ | Nacalai Tesque | 31213-15 | 144-55-8 | 4.2 |
| D-Glucose | FUJIFILM Wako | 049-31165 | 50-99-7 | 5.6 |
| Phenol Red | Nacalai Tesque | 26807-92 | 143-74-8 | 0.02 |

*EGTA Solution*

| **Component** | **SOURCE** | **IDENTIFIER** | **CAS** | **CONCENTRATION (mM)** |
| --- | --- | --- | --- | --- |
| NaCl | FUJIFILM Wako | 191-01665 | 7647-14-5 | 136.9 |
| KCl | Nacalai Tesque | 28514-75 | 7447-40-7 | 5.4 |
| Na_2_HPO_4_.12H_2_O | FUJIFILM Wako | 193-02845 | 10039-32-4 | 0.3 |
| KH_2_PO_4_ | Nacalai Tesque | 2871-55 | 7778-77-0 | 0.4 |
| NaHCO_3_ | Nacalai Tesque | 31213-15 | 144-55-8 | 4.2 |
| D-Glucose | FUJIFILM Wako | 049-31165 | 50-99-7 | 5.6 |
| Phenol Red | Nacalai Tesque | 26807-92 | 143-74-8 | 0.02 |
| HEPES | Nacalai Tesque | 17514-15 | 7365-45-9 | 10 |
| EGTA | Nacalai Tesque | 15214-34 | 67-42-5 | 0.5 |

*Collagenase Solution*

| **Component** | **SOURCE** | **IDENTIFIER** | **CAS** | **CONCENTRATION (mM)** |
| --- | --- | --- | --- | --- |
| NaCl | FUJIFILM Wako | 191-01665 | 7647-14-5 | 136.9 |
| KCl | Nacalai Tesque | 28514-75 | 7447-40-7 | 5.4 |
| Na_2_HPO_4_.12H_2_O | FUJIFILM Wako | 193-02845 | 10039-32-4 | 0.3 |
| KH_2_PO_4_ | Nacalai Tesque | 2871-55 | 7778-77-0 | 0.4 |
| MgSO_4_.7H_2_O | Wako Pure Chemical Industries, Ltd. | 131-00405 | 10034-99-8 | 0.8 |
| CaCl_2_ | Nacalai Tesque | 06729-55 | 10043-52-4 | 5 |
| NaHCO_3_ | Nacalai Tesque | 31213-15 | 144-55-8 | 4.2 |
| D-Glucose | FUJIFILM Wako | 049-31165 | 50-99-7 | 5.6 |
| Phenol Red | Nacalai Tesque | 26807-92 | 143-74-8 | 0.02 |
| HEPES | Nacalai Tesque | 17514-15 | 7365-45-9 | 10 |
| Collagenase | Sigma-Aldrich | C7657 | 9001-12-1 | *) 5 mg/ 40mL |

*) Concentration expressed as wt/vol. of collagenase solution

**Supplementary Table 3.** List of the rare sugars used in the study.

| **REAGENT** | **SOURCE** | **IDENTIFIER** | **CAS** |
| --- | --- | --- | --- |
| L-Sorbitol | Tokyo Chemical Industry | S0388 | 6706-59-8 |
| L-Xylose | Tokyo Chemical Industry | X0021 | 609-06-3 |
| L-(+)-Lyxose | Tokyo Chemical Industry | L0153 | 1949-78-6 |
| L-(+)-Arabinose | Tokyo Chemical Industry | A0515 | 5328-37-0 |
| L-Ribose | Tokyo Chemical Industry | R0068 | 24259-59-4 |
| L-Arabitol | Tokyo Chemical Industry | A0518 | 7643-75-6 |
| L-Talose | Tokyo Chemical Industry | T1767 | 23567-25-1 |
| L-Gulose | Tokyo Chemical Industry | G0239 | 6027-89-0 |
| L-Allulose | Tokyo Chemical Industry | P1778 | 16354-64-6 |
| L-Tagatose | Tokyo Chemical Industry | T2535 | 17598-82-2 |
| L-Sorbose | Tokyo Chemical Industry | S0066 | 87-79-6 |
| L-Talitol | Tokyo Chemical Industry | T2536 | 60660-58-4 |
| L-Glucose | Tokyo Chemical Industry | G0226 | 921-60-8 |
| L-Fructose | Tokyo Chemical Industry | F0317 | 7776-48-9 |
| L-Mannitol | Nagara Science | NS403103 | 643-01-6 |
| D-Erythritol | Tokyo Chemical Industry | E0021 | 149-32-6 |
| D-Maltitol | Tokyo Chemical Industry | M0797 | 585-88-6 |
| D-Mannitol | Tokyo Chemical Industry | M0044 | 69-65-8 |
| D-Sorbitol | Tokyo Chemical Industry | S0065 | 50-70-4 |
| D-Xylitol | Tokyo Chemical Industry | A2635 | 53448-53-6 |
| D-Erythrose | Tokyo Chemical Industry | E0022200MG | 583-50-6 |
| D-Threose | Tokyo Chemical Industry | T3649; CAS: | 95-43-2 |
| D-Xylose | Tokyo Chemical Industry | X0019; | 58-86-6 |
| D-Lyxose | Tokyo Chemical Industry | L0073 | 1114-34-7 |
| D-Arabinose | Tokyo Chemical Industry | A0513 | 10323-20-3 |
| D-Ribose | Tokyo Chemical Industry | R0025 | 50-69-1 |
| D-Arabitol | Tokyo Chemical Industry | A0516 | 488-82-4 |
| D-Ribitol | Tokyo Chemical Industry | R0249 | 130-40-5 |
| D-Allose | Tokyo Chemical Industry | A1488 | 2595-97-3 |
| D-Talose | Tokyo Chemical Industry | T0869 | 2595-98-4 |
| D-Allulose | Tokyo Chemical Industry | P1699 | 551-68-8 |
| D-Tagatose | Tokyo Chemical Industry | T1501 | 87-81-0 |
| D-Sorbose | Tokyo Chemical Industry | S0390 | 3615-56-3 |
| D-Talitol | Tokyo Chemical Industry | T1398 | 643-03-8 |
| D-Iditol | Tokyo Chemical Industry | I0724 | 25878-23-3 |
| D-Galactitol | Tokyo Chemical Industry | G0005 | 608-66-2 |
| D-Allitol | Toronto Research Chemicals | I821250 | 499-40-1 |
| D-Gulose | Santa Cruz Biotechnology | sc-285364 | 4205-23-6 |
| D-Allitol | Santa Cruz Biotechnology | sc-220552 | 27299-12-3 |
| D-Gulitol | Nacalai Tesque | 32021-82 | 50-70-4 |
| D-Ribulose | Cayman Chemical Company | 18893 | 488-84-6 |
| D-Altrose | FUJIFILM Wako | QB-0255 | 1990-29-0 |
| D-Lactitol | FUJIFILM Wako | 120-04092 | 81025-04-9 |
| D-Glucose | FUJIFILM Wako | 049-31165 | 50-99-7 |
| D-Fructose | FUJIFILM Wako | F792500 | 57-48-7 |
| D-Erythrulose | Sigma-Aldrich | 68781-10MG | 496-55-9 |
| D-Threitol | Sigma-Aldrich | 377619 | 2418-52-2 |
| D-Xylulose | Sigma-Aldrich | X4625 | 551-84-8 |
